# Guidance to inform research recruitment processes for studies involving critically ill patients

**DOI:** 10.1177/17511437231197293

**Published:** 2023-09-08

**Authors:** Kerry Woolfall, Katie Paddock, Megan Watkins, Anna Kearney, Katie Neville, Lucy Frith, Ingeborg Welters, Carrol Gamble, John Trinder, Natalie Pattison, Catherine White, Stephen Brett, Steve Dilworth, Mike Ross, Paul Mouncey, Kathy Rowan, Angus Dawson, Clive Collet, Tim Walsh, Bridget Young

**Affiliations:** 1 Department of Public Health, Policy and Systems, Institute of Population Health, University of Liverpool, Liverpool, UK; 2 Faculty of Health and Education, School of Childhood, Youth and Education Studies, Manchester Metropolitan University, Manchester, UK; 3 National Collaborating Centre for Mental Health, Royal College of Psychiatrists, London, UK; 4 Department of Health Data Science, Institute of Population Health, University of Liverpool, Liverpool, UK; 5 Centre for Social Ethics and Policy, University of Manchester, Manchester, UK; 6 Institute of Life Course and Medical Science, University of Liverpool and Department of Critical Care, Liverpool University Hospitals NHS Foundation Trust, Liverpool, UK; 7 South Eastern Health & Social Services Trust, Ulster Hospital, Belfast, UK; 8 University of Hertfordshire and East & North Herts NHS Trust, Stevenage, UK; 9 ICU Steps, London, UK; 10 Imperial College London and Imperial College Healthcare NHS Trust, London, UK; 11 ICU Steps, PPI Partner, UK; 12 PPI Partner, UK; 13 Intensive Care National Audit & Research Centre, London, UK; 14 Centre for Biomedical Ethics, National University of Singapore, Singapore; 15 Health Research Authority, London, UK; 16 University of Edinburgh, Scotland, UK

**Keywords:** Guidance, clinical research, recruitment, consent, critical care

## Abstract

Clinical research in intensive care units (ICUs) is essential for improving treatments for critically ill patients. However, invitations to participate in clinical research in this situation pose numerous challenges. Studies are frequently initiated within a narrow time window when patients are often unconscious and unable to consent. Consultations or consent discussions must therefore be held with consultees or representatives, usually the patient’s relatives. Conversations about research participation in this setting may be difficult, as relatives are often overwhelmed and may feel uneasy about making decisions on behalf of their relatives. In some circumstances, legislation allows doctors to act as consultees or representatives to enrol patients in research. However, there is little good quality evidence on UK stakeholders’ perspectives to inform how recruitment is carried out in ICU studies. The Perspectives Study collected evidence on the views of over 1400 stakeholders, including patients, relatives and healthcare practitioners, many of whom had first-hand experience of ICU treatment and research. This evidence was used to inform good practice guidance on recruitment of critically ill patients to research. Established social science methods and empirical ethics were employed to reflect the interests of stakeholders and justify recommendations. This guidance aims to bridge the gap between the legal frameworks and the realities of ICU studies and to ensure that research recruitment processes reflect the views of patients and families. Researchers and an expert Advisory Group brought different perspectives to interpreting the evidence to develop the guidance. In this article we present guidance for future ICU studies.

## Introduction

Clinical research is important for improving the health, care and treatments for critically ill patients.^1^ The process of recruiting and seeking consent within the context of critical care differs to many other clinical settings. Whilst informed consent has been considered a hallmark of ethical research, it is often not possible to seek consent prospectively within the context of critical care when studies are initiated in a narrow time window and patients do not have the capacity to make a decision and there is no time or opportunity to involve a family representative in those decisions due to the urgent need to provide treatment. Lack of capacity mainly arises from sedation, but could also be due to issues such as confusion, delirium, severe pain, inability to communicate and anxiety.^2^ Even when a patient has capacity, conversations about research participation may seem peripheral to patients and their family members who are likely to feel overwhelmed, uncomfortable about making decisions in this context and unable to comprehend study information.

Alternative processes are permitted for recruiting and seeking consent for critical care patients that aim to balance the rights of critically ill patients with the need to conduct research to improve outcomes.^3^ In 2018/19, the UK National Institute of Health Research Clinical Research Network supported 104 critical care studies, recruiting 41,045 patients. More recently during the COVID-19 pandemic the network supported the delivery of national and international studies to develop knowledge, diagnostics and treatments for COVID-19. Such activity is governed by legislation including the Human Rights Act (1998) and the Mental Capacity Act (2005).^4,5^ While resources on legal frameworks are available to ICU research teams, accessing and implementing these can be challenging in the reality of clinical practice. Moreover, ensuring that research recruitment processes are ethical, as well as patient and family centred, is beyond the scope of legal frameworks. Healthcare practitioners will inevitably have to bridge these gaps when recruiting patients to studies, and the good practice guidance we report here aims to help healthcare practitioners in doing so. It is based on evidence on the perspectives of patients, family members and healthcare practitioners regarding ICU research recruitment processes when a critically ill patient needs immediate clinical care, including when patients lack capacity or when patients die after study enrolment.

## Methodology

We summarise the methods used to develop the guidance below. Further details of empirical research methodology are outlined in our related publications.^6,7^

The PERSPectives on Enhancing Consent and recruiTment in IntensiVe carE Studies (The Perspectives Study) explored the views and experiences of patients, their family members, and healthcare practitioners regarding recruitment procedures of studies that take place in ICUs. The Perspectives study began in September 2016 and concluded in May 2020.

**Figure 1.**
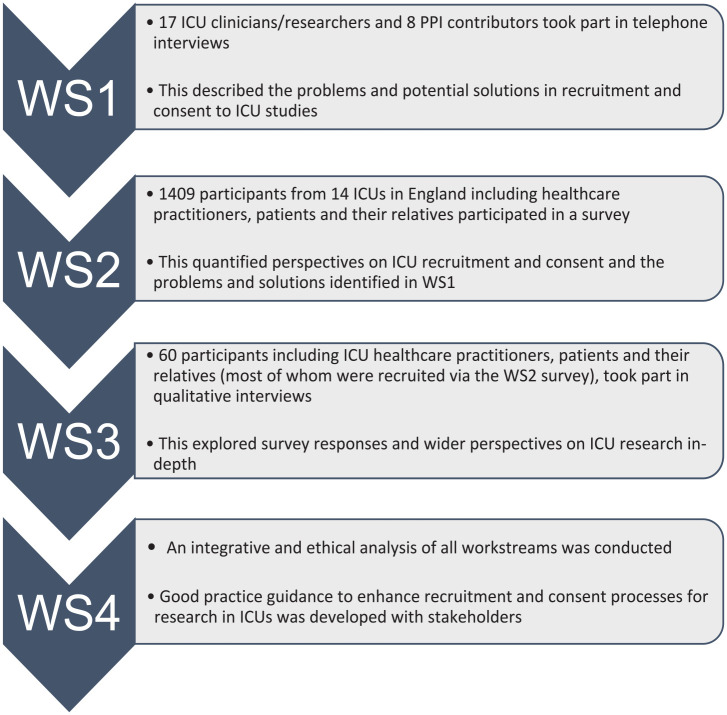
Flowchart showing stages of research (Workstreams 1–4).

Established social science methods comprising surveys, qualitative interviews and ethical analysis were used across three interrelated empirical work streams (see Figure 1). Stakeholders included those with recent first-hand experience of ICU treatment or research participation, including patients, family members (some of whom were bereaved), healthcare practitioners (nurses, doctors, allied health professionals and pharmacists), clinical and non-clinical researchers, and Patient and Public Involvement (PPI) advocates. In brief, work stream 1 involved qualitative interviews with 17 ICU clinicians/researchers and 8 patient and public involvement (PPI) contributors with experience of working on ICU studies. Findings informed the development of a work stream 2 survey involving 1409 patients, family members and healthcare practitioners in 14 ICUs across England. In work stream 3 we purposively sampled 60 individuals for qualitative interview (via workstream 2) to explore their survey responses and their wider perspectives on ICU research in more depth.

To develop the recommendations, in a fourth work stream we considered the findings from the three empirical work streams in light of ethical principles such as justice, autonomy and non-maleficence and wider literature.^8–12^ A 1-day meeting (Liverpool, 2019) was convened to review and develop the guidance involving 28 key stakeholders including ICU practitioners, ethicists, former ICU patients and family members with ICU experience and members of the study team. Post-meeting guidance drafts were developed in collaboration with our expert advisory group which included patient partners (SD, MR, CW), ICU doctors (SB, JT, TW, IW), nurses (NP), researchers (KW, KP, KN, BY, CG, KR, PM) and ethicists (LF, AD, CC).

The Perspectives study provided new evidence which confirmed the acceptability of most current critical care recruitment, consultation and consent processes. The following sections provide an overview of key recommendations from the Perspectives Study, highlighting new insights to inform adult ICU research recruitment. The full guidance is available at: https://www.liverpool.ac.uk/media/livacuk/iphs/1healthservicesresearch/Perspectives_guidance_Version,1.0,02.04.2020.pdf

## The good practice guidance

### What type of ICU studies is this guidance for?

This guidance has been developed for UK research studies in both emergency and non-emergency adult critical care situations. However, it may also benefit those involved in the design, conduct and review of ICU research internationally. Some recommendations in the guidance are only applicable to studies where patients are randomised at the individual rather than cluster (group) level. All research should be conducted in accordance with appropriate legislation that govern the jurisdiction where the research takes place. Study protocols and documents should be approved by a research ethics committee.

#### What should I consider at the study design stage?

**Figure 2.**
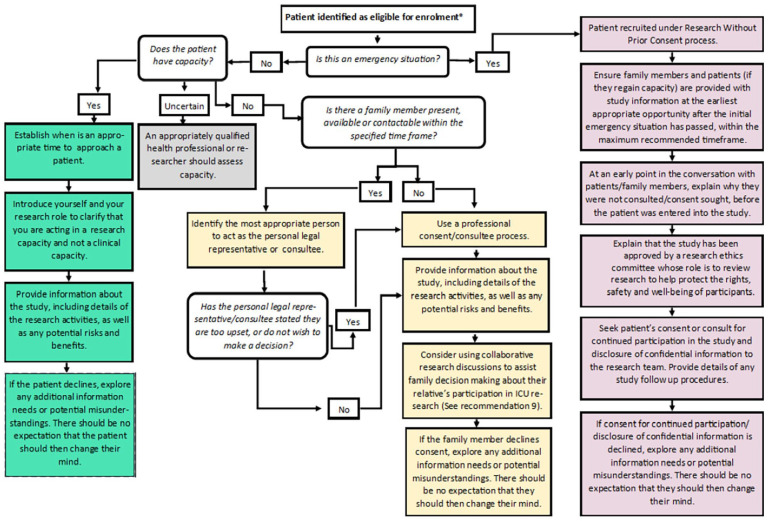
Flow chart of decision points in recruitment and consent to critical care studies.

Research teams should consider which recruitment processes are most appropriate in terms of expected patient capacity and the nature of the proposed research activities. This should include a review of the setting, maximum anticipated time window for approaching patients or family members, and the expected capacity of eligible patient population. Research teams should ensure adequate resourcing is in place, including staffing. The flowchart in Figure 2 shows the decision points in recruitment and consent to critical care studies to assist the study design.

Consider conducting pre-study research (e.g. a feasibility study) and ensure Patient and Public Involvement (PPI) contributors can inform all aspects of the study, including the design, research questions and the proposed recruitment processes.

Delaying PPI until funding is awarded^6^ and allocation of PPI roles that focus mainly on steering group attendance can limit the opportunity for PPI contributors to inform studies.^13^ Therefore, at an early point in planning, it is important to actively involve and support PPI contributors (who have relevant experience of critical illness as a patient or family member) to input to the design of study processes, procedures, information materials, the selection and measurement of study outcomes and dissemination of the findings.

Versions of written study information should be tailored to patients, to their families/friends who are consulted about the study (referred to as personal representatives in Clinical Trials of Investigational Medicinal Products (CTIMPs) and personal consultees (in non-CTIMPs), and to practitioners who are not connected with the study (referred to as professional representatives (CTIMPs) or consultees (non-CTIMPs).^7,14^ This written information should include a short summary section to assist the tailoring of information to the capacity of patients and personal representatives/consultees and avoid information overload. Hard copies of documents can be lost during the ICU stay and patients/family members often wish to keep all information about their/the patient’s ICU stay.^7^ Study teams should provide the option of access to online participant information materials (e.g. via links in patient ICU diaries to study websites). Also consider using ICU patient diaries to record studies that patients have been recruited to so that they can access details of these later if they wish and when they feel able to.

#### When should I approach patients who have capacity to discuss research?

Patients, families and healthcare practitioners in the Perspectives study supported seeking informed consent for research participation from the patient when possible,^6^ which is consistent with legal frameworks and with the principle of respect for patient autonomy.^8^

Staff should introduce themselves to patients, clarify that they are acting in a research capacity and acknowledge the difficulty of the situation. Before the research is discussed, staff should ensure the patient has had an update about their condition from clinical staff. It is important to consider how information about the study could be tailored to the capacity and understanding of the patient. Research staff should be prepared to repeat information because patients may not always fully comprehend information when first given. This may involve going back to the patient on a number of occasions unless the patient indicates they do not want to be consulted further. Staff should also consider using multimedia resources for example, animations or videos to support information provision and supplement written information leaflets and discussion.

In these circumstances, research staff should draw on their professional judgement to establish when is an appropriate time to approach a patient. They might also consult the clinical care team and/or family members, and at an early point in the conversation, ask the patient if they feel able to have a conversation about research.

#### When and whom should I approach to discuss research if a patient lacks capacity?

When a patient lacks capacity to consent for themselves, most patients, family members, and practitioners consider it acceptable for a family member to decide about research participation on behalf of incapacitated patients. However, family members will have had little experience of medical research and personal consultee/consent processes may seem burdensome for some family members in the context of critical illness.^7^

Research staff should approach family members to discuss research at the earliest appropriate opportunity. Also consider whether there may be additional factors causing stress at that moment in time, such as whether the family recently arrived in the ICU for the first time, if the patient’s condition has recently deteriorated, or they have recently received bad news. In such circumstances consider waiting for a better time to approach (research window permitting).

When appropriate timing is established, identify who is the most appropriate person to act as the patient’s personal representative for written consent, or their personal consultee. This is likely to be the patients’ next of kin, close friend or family member. The aims of the research and staff roles should be clarified and the impact of the study on the patient’s care explained.

Legislation and guidance present research discussions with family members as separate to research discussions involving staff. However, collaborative research discussions between families and the research team could assist families in making decisions about their relative’s participation in ICU research, and help the family in establishing what is in that patient’s best interests (according to the UK Mental Capacity Act [2004]). These discussions do not necessarily have to include all staff and family members in the room at the same time, rather whatever is practically possible. Such discussions could also help to identify who should act as the consultee or representative (e.g. if there is difficulty identifying who is next of kin). This collaborative discussion could include the professional consultee and/or a member of the clinical care team who is independent from the research. When family members do not reach agreement, then it is appropriate to offer to help them come to a decision by responding to any queries or concerns. Consider providing the family with the option of a professional consultee to decide on their behalf. If a family member declines consent or advises against study participation, identify any additional information needs or misunderstandings. There should be no expectation that the family member should then change their mind.

#### When should I use a professional consent or professional consultee process?

When a patient lacks capacity, all reasonable efforts to contact family members to discuss the research should be made within the study recruitment window. Family members emphasised the importance of feeling involved in decisions relating to the patient.^7^ Face-to-face discussions with families to inform these decisions are preferable. However, telephone calls are preferred to no discussion with families at all.

Where reasonable efforts have been made to reach the family but they cannot be contacted within the recruitment window, or when contacted families have stated they are too upset or do not wish to make a decision, patients, families and healthcare practitioners supported the use of professional consent or professional consultee process in recruitment to ICU studies.

When patients have been enrolled in a study using a professional consent or consultee process, research staff should inform family members about the patient’s participation in the study at the earliest appropriate opportunity. As well as providing them with information about the study, they might also explain the professional consent/consultee process and why this was used, and how the patient will be provided with study information if/when they regain capacity.

#### What should I consider in an emergency situation when a patient has been entered into a trial without their prior consent?

In an emergency situation where there is no time for personal or professional consent, patients can be entered into a study without prior consent or consultation with family members.^15^ Recruitment to research without prior consent, often referred to as ‘deferred consent’, requires approval by a Research Ethics Committee. In such situations, the Perspectives study identified the importance of a strategy for approaching and structuring discussions with patients (if they regain capacity) and/or family members about studies after the initial emergency has passed. Box 1 provides key points to consider.

**Box 1.** Points to consider when discussing research without prior consent with patients and/or family members.

- Ensure family members and patients (if they regain capacity) are provided with study information at the earliest appropriate opportunity after the initial emergency situation has passed, within a maximum recommended timeframe. - Firstly acknowledge the difficulty of their situation and what they have been through. - At an early point in the conversation with patients and/or family members, research staff should explain why consent was not sought, or why they were not consulted, before the patient was entered into the study. This should include explaining that it was not possible to hold the conversation before study recruitment because the patient needed immediate clinical care, which could not be delayed. - Explain that the decision reached will not impact on the quality of their/their relatives’ care. - If the patient regains capacity after the emergency has passed, seek their consent or consult with them about their continued participation in the study and use data and/or disclosure of confidential information within the study. Provide details of any study follow-up procedures (if applicable). - If the patient or family member declines consent for continued participation etc, explore any additional information needs or potential misunderstandings. There should be no expectation that they should then change their mind.

#### What should I consider when a patient dies before the study is discussed?

Sometimes patients enrolled in studies under an emergency or professional consent/consultee process die before the study is discussed with family members. There will, therefore, be situations where bereaved family members are unaware that their relative has participated in a study and that their data will be included. Although there is no legal obligation to discuss research participation with bereaved family members (unless the study involves disclosure of confidential information), there are indications from the Perspectives Study and previous research with bereaved parents in paediatric critical care studies,^16,17^ that families wish to be informed about their loved one’s research participation. The recommendations in Box 2 reflect this and include options to help research staff identify the most appropriate approach for each individual family. The chosen procedure should be detailed in the study protocol and approved by a research ethics committee. Recommendations are tentative until further research is conducted exploring the views of bereaved family members and ICU researchers who have experienced the recommended approaches.

**Box 2.** Options to consider when a patient has died.

**Option 1: Approach family members with study information before they leave hospital.** - Discuss the study and provide information before family members leave hospital. However, only approach family members at this point if it is believed to be appropriate and local bereavement guidance has already been followed. **Option 2: Contact family members to arrange a face-to-face discussion.** - If it is not thought appropriate to discuss the study before family members leave the hospital, consult with colleagues to identify an appropriate time to contact family members by a personalised letter or phone call to provide the option of a face-to-face visit to discuss research. - If a letter is used, these should be written at the trial design stage in close consultation with bereaved family members, bereavement specialists, or relevant groups. The letter should be personalised, signed by a clinician (known to the family if possible), include a named contact and telephone number, and emphasise that a face-to-face meeting is optional. If family members do not wish to have a face-to-face meeting, offer to send written information about the trial and provide contact details in case the family want to discuss the research at a later date. - During face-to-face discussions, explore family members’ views and understanding of the study and explain why they were not consulted/their consent was not sought prior to the patient’s enrolment, so that any concerns can be addressed. - Seek consent/consult family members for disclosure of confidential information to the research team. Be prepared to respond to family members who are concerned that study participation may have contributed to their relative’s death

Staff should use their professional judgement to inform when and how to approach and discuss research with bereaved family members. The approach should complement bereavement guidance at each participating hospital. While all research discussions should be personalised and conducted with sensitivity, this is especially true of discussions with bereaved family members. The Principal Investigator and/or medical/nursing staff known to the family should establish which of the following options is most appropriate for each family.

#### How can I raise awareness about ICU research

To help improve understanding about alternative consent processes in ICU research there is a need to raise awareness about their use and acceptability amongst the public and clinical staff. Staff training and research focussed newsletters as well as public facing social media, information leaflets, poster and animations can be used to publicise how the hospital conducts research to help save and improve critically ill patients’ lives. This might include publicising specific examples of published ICU research to help convey the message and their impact on clinical care. For studies conducted in Critical Care that involve recruitment of patients without prior informed consent and/or consent/consultation with family members, briefly explain: study aims, inclusion criteria, why informed consent cannot be sought prospectively and advise patients and family members that they may be approached by a research nurse about a study. Provide details of where patients and family members can access further information, including study findings when available.

Finally, ensure patients and families members are provided with the opportunity to access plain language summaries of findings online. Many studies take several years for the findings to become available, so patients’ and families’ expectations about when the study results will be available should be managed in line with anticipated timescales.

## Conclusions

Perspectives study guidance helps to bridge the gap between the legal frameworks and the realities of ICU studies for all involved and ensure that research processes are patient and family centred. Some of the recommendations add to current practice in studies conducted in critical care, while other recommendations confirm the acceptability to stakeholders of current practice in critical care, and of wider guidance on research in healthcare settings. The full Perspectives Study guidance and supplementary resources can be found at: https://www.liverpool.ac.uk/population-health/research/groups/perspectives/resources/

## References

[1] Reay H Arulkumaran N Brett SJ. Priorities for future intensive care research in the UK: results of a James Lind alliance priority setting partnership. J Intensive Care Soc 2014; 15: 288–296. 10.1177/1751143715609954 PMC5606390 28979474

[2] El-Menyar A Asim M Latifi R , et al. Research in emergency and critical care settings: debates, obstacles and Solutions. Sci Eng Ethics 2016; 22: 1605–1626. 26602908 10.1007/s11948-015-9730-5

[3] Honarmand K Belley-Cote EP Ulic D , et al. The deferred consent model in a prospective observational study evaluating myocardial injury in the Intensive Care Unit. J Intensive Care Med 2018; 33: 475–480. 29991343 10.1177/0885066616680772

[4] Health Research Authority. The mental capacity act, https://www.hra.nhs.uk/planning-and-improving-research/policies-standards-legislation/mental-capacity-act/ (2020, accessed 18 November 2023).

[5] Jones S Gill P Kenkre J. Nurse managed patient focused assessment and care: a grounded theory of qualified nurses in acute and critical care settings assessing the mental capacity of adult patients. J Clin Nurs 2020; 29: 1254–1266. 31951067 10.1111/jocn.15188

[6] Paddock K Woolfall K Frith L , et al. Strategies to enhance recruitment and consent to intensive care studies: a qualitative study with researchers and patient-public involvement contributors. BMJ Open 2021; 11: e048193. 10.1136/bmjopen-2020-048193 PMC8461270 34551943

[7] Paddock K Woolfall K Kearney A , et al. Learning from stakeholders to inform good practice guidance on consent to research in intensive care units: a mixed-methods study. BMJ Open 2022; 12: e066149. 10.1136/bmjopen-2022-066149 PMC9664286 36375987

[8] Beauchamp T Childress J. Principles of biomedical ethics. Oxford: Oxford University Press, 2019.

[bibr9-17511437231197293] Gunson D. Solidarity and the universal declaration on bioethics and human rights. J Med Philos 2009; 34: 241–260. 19387000 10.1093/jmp/jhp022

[bibr10-17511437231197293] Kottow M. The battering of informed consent. J Med Ethics 2004; 30: 565–569. 15574446 10.1136/jme.2003.002949 PMC1733983

[bibr11-17511437231197293] Molewijk B Frith L. Empirical ethics: who is the don quixote? Bioethics 2009; 23: ii–iv. 10.1111/j.1467-8519.2009.01707.x 19338519

[12] Young R. Informed consent and patient autonomy. In: HS Kuhse (ed.) A companion to bioethics. Malden: Blackwell Publishing, 2012, pp.530–540.

[13] Gamble C Dudley L Allam A , et al. Patient and public involvement in the early stages of clinical trial development: a systematic cohort investigation. BMJ Open 2014; 4: e005234. 10.1136/bmjopen-2014-005234 PMC4120322 25056972

[14] Shepherd V. Research involving adults lacking capacity to consent: the impact of research regulation on ‘evidence biased’ medicine. BMC Med Ethics 2016; 17: 55. 27609355 10.1186/s12910-016-0138-9 PMC5016956

[15] Health Research Authority. Research in emergency settings, https://www.hra.nhs.uk/planning-and-improving-research/policies-standards-legislation/research-emergency-settings/ (2020, accessed 18 November 2023).

[16] O’Hara CB Canter RR Mouncey PR , et al. A qualitative feasibility study to inform a randomised controlled trial of fluid bolus therapy in septic shock. Arch Dis Child 2018; 103: 28–32. 28847877 10.1136/archdischild-2016-312515 PMC5754873

[17] Woolfall K Frith L Dawson A , et al. Fifteen-minute consultation: an evidence-based approach to research without prior consent (deferred consent) in neonatal and paediatric critical care trials. Educ pract ed 2016; 101: 49–53. 10.1136/archdischild-2015-309245 PMC4752644 26464416

